# ^111^In-cetuximab as a diagnostic agent by accessible epidermal growth factor (EGF) receptor targeting in human metastatic colorectal carcinoma

**DOI:** 10.18632/oncotarget.3968

**Published:** 2015-05-14

**Authors:** Ying-Hsia Shih, Cheng-Liang Peng, Shin-Yu Lee, Ping-Fang Chiang, Cheng-Jung Yao, Wuu-Jyh Lin, Tsai-Yueh Luo, Ming-Jium Shieh

**Affiliations:** ^1^ Institute of Biomedical Engineering, College of Medicine and College of Engineering, National Taiwan University, Taiwan; ^2^ Institute of Nuclear Energy Research, Atomic Energy Council, Taoyuan, Taiwan; ^3^ Department of Oncology, National Taiwan University Hospital and College of Medicine, Taiwan; ^4^ Institute of Radiological Science, Central University, Taiwan; ^5^ Division of Gastroenterology, Department of Internal Medicine, Wan Fang Hospital, Taipei Medical University, Taipei, Taiwan

**Keywords:** colorectal adenocarcinoma, EGF receptor, cetuximab, nano-SPECT/CT

## Abstract

Colorectal adenocarcinoma is a common cause death cancer in the whole world. The aim of this study is to define the ^111^In-cetuximab as a diagnosis tracer of human colorectal adenocarcinoma. In this research, cell uptake, nano-SPECT/CT scintigraphy, autoradiography, biodistribution and immunohitochemical staining of EGF receptor were included. HCT-116 and HT-29 cell expressed a relatively high and moderate level of EGF receptor, respectively. The nano-SPECT/CT image of ^111^In-cetuximab showed tumor radiation uptake of subcutaneous HCT-116 xenograft tumor was higher than SW-620. Autoradiography image also showed that tumor of HCT-116 had high ^111^In-cetuximab uptake. Mice that bearing CT-26 *in situ* xenograft colorectal tumors showed similar high uptake *in vivo* and *ex vivo* through nano-SPECT/CT imaging at 72 hours. Metastatic HCT-116/Luc tumors demonstrated the highest uptake at 72 hours after the injection of ^111^In-cetuximab. Relatively, results of ^111^In-DTPA showed that metabolism through urinary system, especially in the kidney. The quantitative analysis of biodistribution showed count value of metastatic HCT-116/Luc tumors that treated with ^111^In-cetuximab had a significant difference (*P* < 0.05) compared with that treated with ^111^In-DTPA after injection 72 hours. Result of immunohistologic staining of EGF receptor also showed high EGF receptor expression and uptake in metastatic colorectal tumors. In summary, we suggested that ^111^In-cetuximab will be a potential tool for detecting EGF receptor expression in human metastatic colorectal carcinoma.

## INTRODUCTION

Colorectal adenocarcinoma is a common cause of cancer death in the whole world. Patients of colon cancer often have no symptom in early stage, but once diagnosed that always have poor treatment effect in the third or fourth late stage. [1–4] Especially, the metastasis of colorectal cancer always caused the worst symptoms. [5] However, early screening can reduce mortality of colorectal cancer.

The over expression of epidermal growth factor (EGF) receptor was found in some patients of colon cancer. [6] Cetuximab had been reported that could specifically target cells expressing EGF receptor for treatment. [7–9] EGF receptor is associated with the control of cell survival, cell cycle, angiogenesis, and other metastasis related signaling pathway including cell migration and cell invasion. [7–13] Cetuximab is a chimeric monoclonal IgG1 antibody that directed against epidermal growth factor and inhibits its activation, which then leading to the inhibition of tumor cell growth and the induction of apoptosis. [14–16] Compared with its affinity to the endogenous substrates, EGF receptors have a 5 to 10 times higher affinity to Cetuximab. [17] It also inhibits the generation of the generation of the angiogenic factor, and blocks endothelial cell migration, thereby reducing tumor angiogenesis and metastasis. [9, 18] Many researches have shown that cetuximab can be used for targeting tumor cells that express the epidermal growth factor receptor, and execute the cytotoxic effect.[14–16, 18] Cetuximab does not bind to HER family. [18] ^111^In (half-life = 2.83 days, *r*-ray = 0.2454 MeV) is usually used in labeling cetuximab for the disease diagnosis, such as head and neck cancer. [19, 20] ^111^In was as an image tracer with the cetuximab antibody in this study.

**Figure 1:**
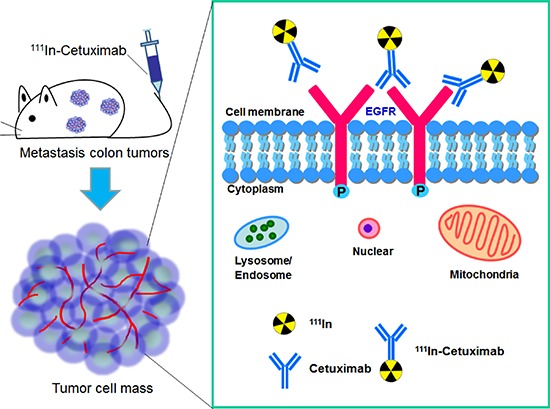
^111^In-Cetuximab synthesis, experimental design and EGF receptor image in human metastasis colorectal carcinoma

In this study, initial we used ^111^In labeled cetuximab to target EGF receptors in subcutaneously induced human colon tumor, and then further applied it for the diagnosis of human metastatic colorectal adenocarcinoma (Figure 1). We considered that ^111^In-cetuximab can be used for the diagnosis of human metastatic colon cancer, and then providing enhanced therapeutic effect.

## RESULTS

### *In vitro* cell uptake of cetuximab

**Figure 2:**
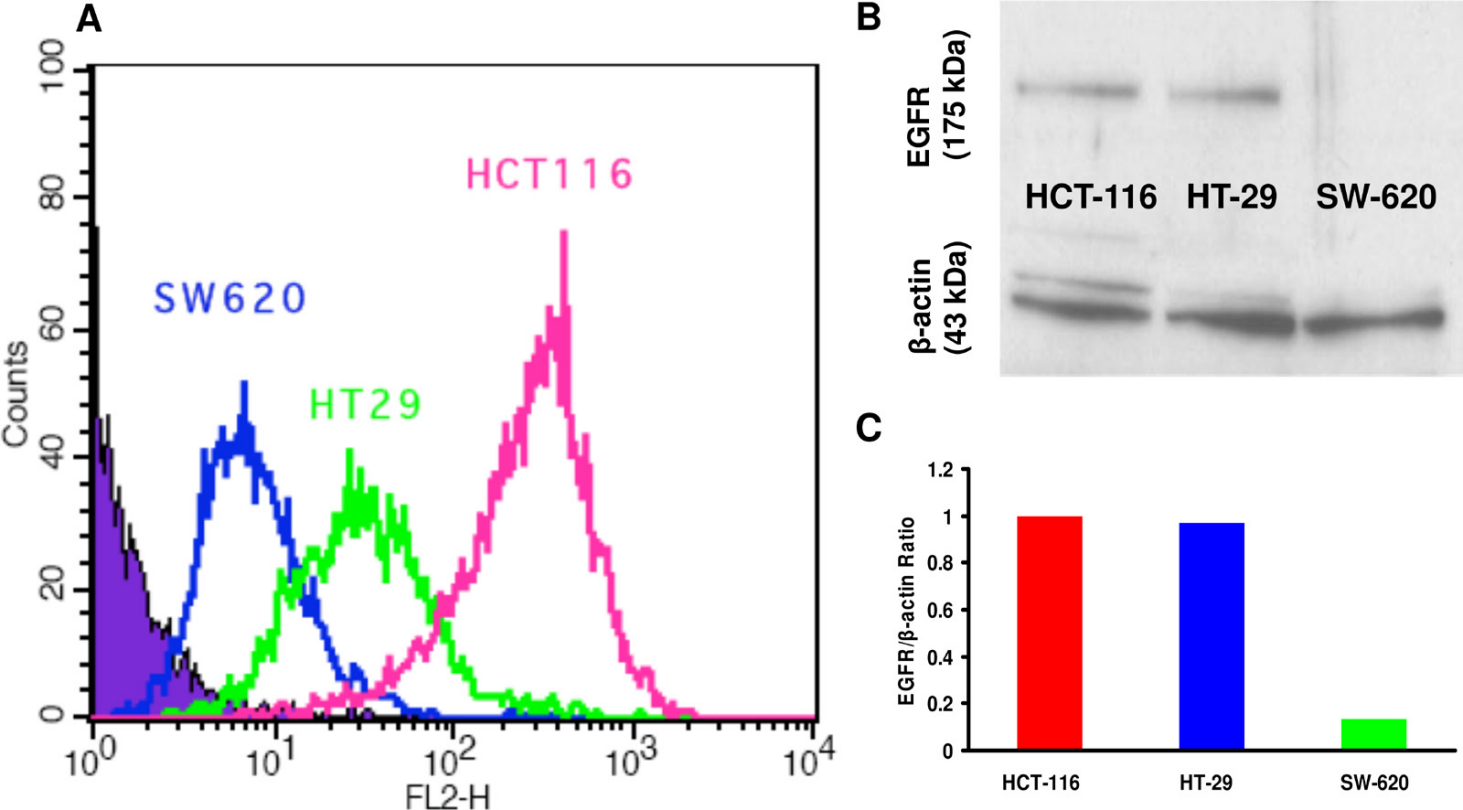
Flow cytometry analysis and western blot of EGF receptor expression in HCT-116, HT-29 and SW-620 colon cancer cell lines

We used cetuximab to target the EGF receptor (EGFR) expressed on different colon cancer cell lines, and the EGF receptor expression profile measured by the flow cytometry was showed in Figure 2A, which suggested that among the three cell lines (HCT-116, HT-29, SW-620) we studied. HCT-116 has a higher EGF receptor expression than HT-29, and SW-620 has the lowest expression level. The results of western blot (Figure 2B) also showed different levels of EGF receptor expression were detected in the colorectal cancer cell lines. The EGF receptor/β-actin radio of HCT-116, HT-29 and SW-620 were 1, 0.97 and 0.13, respectively (Figure 2C). HCT-116, HT-29 cells expressed a relatively high and moderate level of EGF receptor, respectively, while SW-620 expressed a low level of EGF receptor, which was considered as negative in this study.

### Nano-SPECT/CT and autoradiography scintigraphy image of ^111^In-cetuximab in HCT-116 and SW-620 subcutaneous colorectal tumor-bearing mice

**Figure 3:**
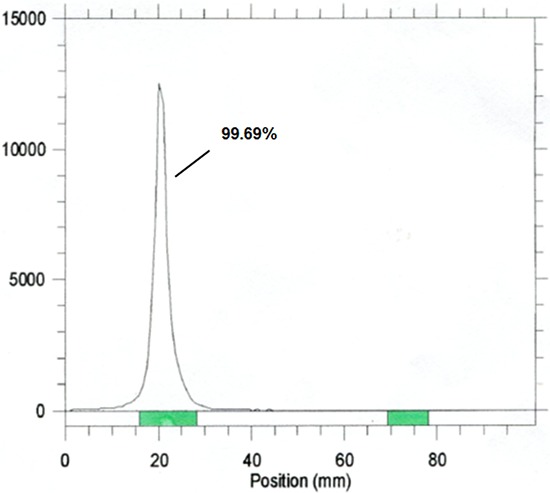
Radiochemistry purity of ^111^In-Cetuximab

**Figure 4:**
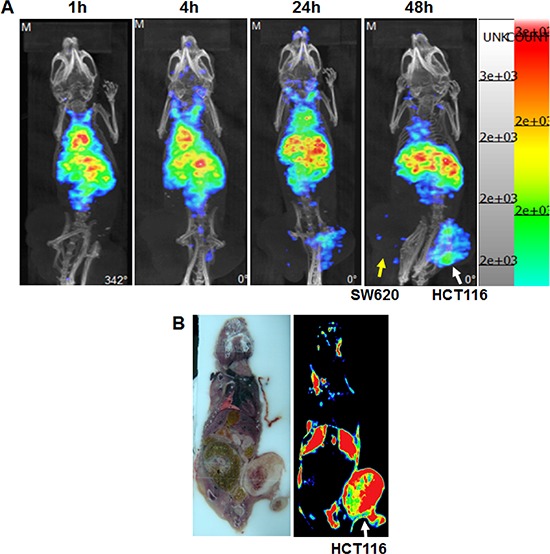
Nano-SPECT/CT **A.** images in HCT-116 and SW-620 subcutaneously tumors images obtained after 1, 4, 24, 48 hours intravenous injection of ^111^In-Cetuximab HCT-116 bearing in right leg, SW-620 bearing in left leg. Autoradiography **B.** images obtained at 48 hours after intravenous injection of ^111^In-Cetuximab.

For all studies in this report, ^111^In-cetuximab with high radiochemistry purity (> 95%) was used (Figure 3). The nano-SPECT/CT image of mice bearing subcutaneous tumors showed that HCT-116 tumor (on the right flank of the mouse) had higher uptake of ^111^In-cetuximab than SW-620 tumor (left flank) 24 hours after the intravenous injection of ^111^In-cetuximab (Figure 4A). Similar to other nanoparticles, it was inevitable that the liver had high uptake due to the reticuloendothelial system (RES) function. HCT-116 tumors demonstrated the highest uptake at 48 hours, while the uptake of SW-620 tumors still remained very low, which was corresponded with the different EGF receptor expression level of HCT-116 and SW-620. Results of autoradiography images also showed that tumor of HCT-116 had higher ^111^In-cetuximab uptake after 72 hours injection of ^111^In-cetuximab (Figure 4B).

### *In vivo* and *ex vivo* nano-SPECT/CT image of ^111^In-cetuximab *in situ* CT-26 colorectal tumor-bearing mice

**Figure 5:**
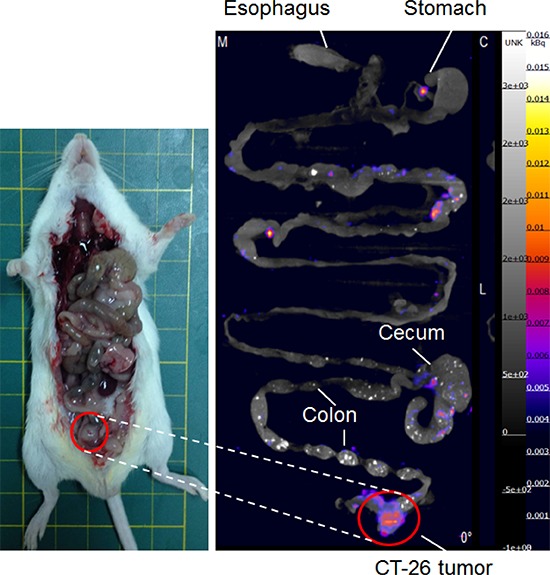
*In vivo* and *ex vivo* Nano-SPECT/CT images of ^111^In-cetuximab *in situ* CT-26 colorectal tumor-bearing mice at 72 hours after intravenous injection of ^111^In-cetuximab *Ex vivo* image was obtained uptake in the colorectal tumor area after ^111^In-cetuximab intravenous injection.

Mice bearing *in situ* CT-26 colorectal tumor showed similar high uptake of ^111^In-cetuximab through *in vivo* and *ex vivo* nano-SPECT/CT imaging (Figure 5). The *in vivo* and *ex vivo* nano-SPECT/CT images indicated that *in situ* CT-26 colorectal tumors had the highest uptake at 72 hours after the intravenous injection of ^111^In-cetuximab. The stomach and cecum had lower ^111^In-cetuximab uptake, and there was no uptake in esophagus and normal colon tissue.

### Nano-SPECT/CT and autoradiography scintigraphy image of ^111^In-cetuximab and ^111^In-DTPA, and the immunohitochemical detection of EGF receptor in HCT-116/Luc metastasis colorectal tumor-bearing mice

**Figure 6:**
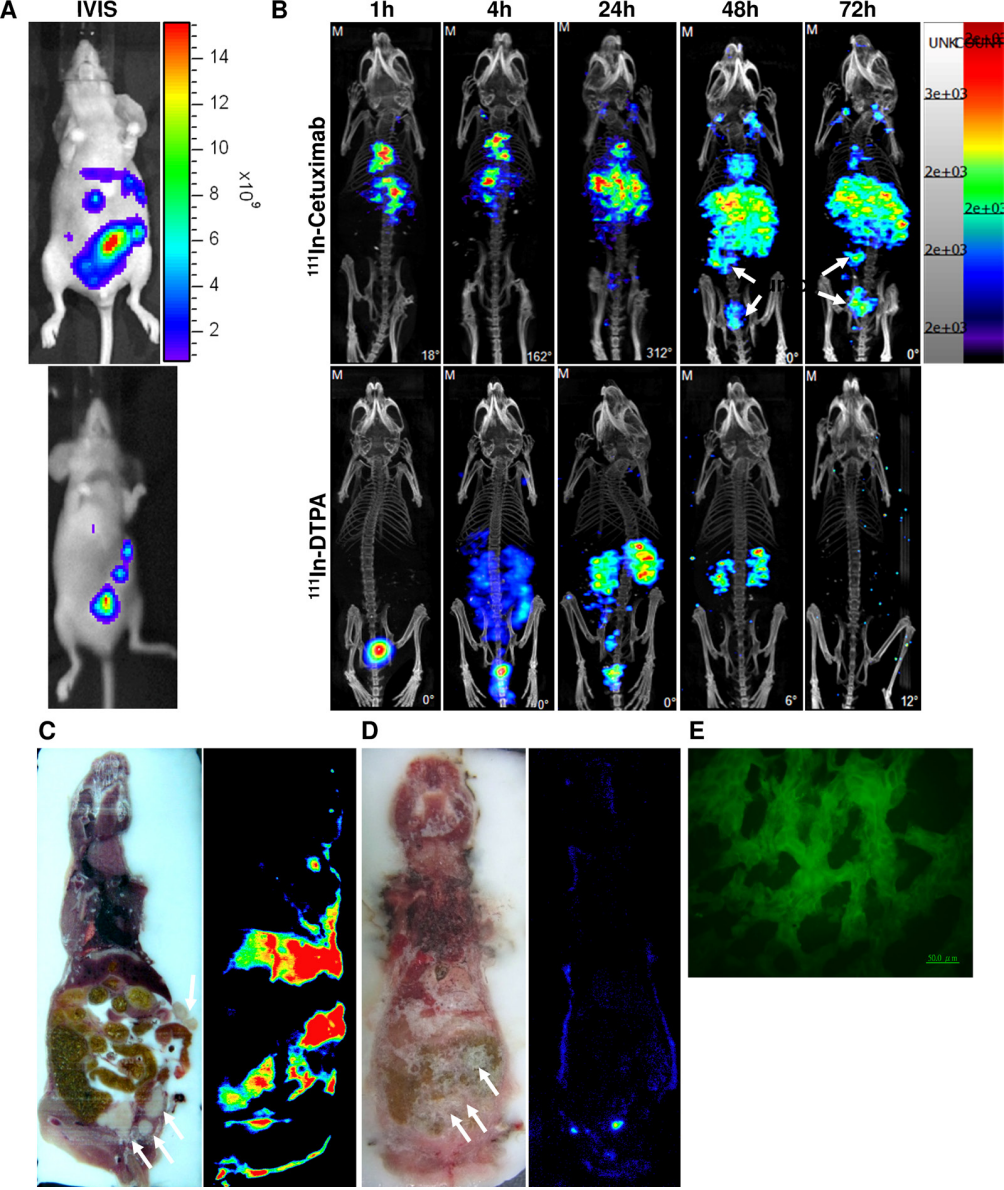
HCT-116/Luc metastatic colorectal tumors (white arrow indicates abdominal colorectal tumors) IVIS images **A.** Nano-SPECT/CT image **B.** and autoradiography obtained at 1, 4, 24, 48, 72 hours after intravenous injection of ^111^In-cetuximab **C.** and ^111^In-DTPA **D.** Autoradiography images obtained at 72 hours after intravenous injection of ^111^In-cetuximab and ^111^In-DTPA. Immunohistologic staining **E.** of EGF receptor in the tumor section.

The IVIS images of mice showed uptake of lucferrin in the tumor region before the administration of ^111^In-cetuximab and ^111^In-DTPA (Figure 6A). Nano-SPECT/CT images showed radiation uptake of ^111^In-cetuximab and ^111^In-DTPA in HCT-116/Luc metastatic tumor at 24 hours after the intravenous injection (Figure 6B), and the highest uptake was observed at 72 hours after injection of ^111^In-cetuximab. There still had a high uptake observed in the liver of mice. On the Contrary, without being uptake by the tumor, ^111^In-DTPA was metabolized through the urinary system, especially in the kidney. 72 hours after the injection, there was no signal of ^111^In-DTPA observed in the nano-SPECT/CT image. Autoradiography image showed that metastatic tumor targeted by ^111^In-cetuximab had higher radiation uptake than ^111^In-DTPA (Figure 6C and 6D). Result of immunohistologic detection of EGF receptor also showed high EGF receptor expression in the metastatic tumor region (Figure 6E).

### Biodistribution of ^111^In-cetuximab and ^111^In-DTPA in HCT-116/Luc metastatic colorectal tumor

**Figure 7:**
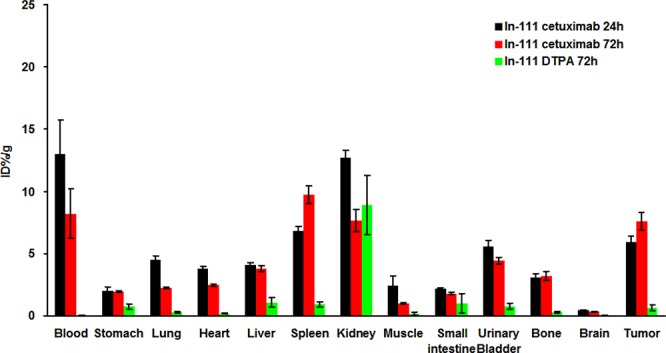
Biodistribution of ^111^In-cetuximab and ^111^In-DTPA in metastatic colorectal tumors and normal organs at 24 and 72 hours after intravenous injection There is a statistically significant difference (*P* < 0.01) between ^111^In-cetuximab and ^111^In-DTPA at 72 hours after injection.

The biodistributions of the ^111^In-cetuximab and ^111^In-DTPA were determined by *γ*-counting, respectively. The results demonstrated that the ^111^In-cetuximab was mainly distributed in the kidney, spleen and tumors (Figure 7). After the injection, most of ^111^In-cetuximab was circulating in the blood within 72 hours. On the other hand, with relatively low accumulation in spleen, small intestine, liver and stomach, ^111^In-DTPA was mainly accumulated in the kidney within 72 hours.

## DISCUSSION

The *in vitro* cellular uptake of cetuximb measured by the flow cytometry and the expression level of EGF receptor confirmed by western blot (Figure 2) demonstrated a similar result and suggested that there were different expression level of EGF receptor between the three human colorectal cancer cell lines: HCT-116 and HT-29 had a high to moderate EGF receptor expression, while the expression level of SW-620 was very low, which could be considered as negative among this three cell lines.

In studies of *in vivo* tumor uptake by using the subcutaneous colorectal tumor-bearing mice model, the nano-SPECT/CT and autoradiography images both revealed higher radiation uptake in HCT-116 tumors than SW-620 tumors at 24 to 48 hours after the intravenous injection of ^111^In-cetuximab (Figure 4). Similar result was also observed in the head and neck cancers. [21] These results also corresponded with that the application of ^111^In-cetuximab targeting demonstrated a higher EGF receptor expression in HCT-116 than in SW-620 cells.

Next, the results of uptake studies using CT-26 colorectal tumor-bearing mice showed that ^111^In-cetuximab could specifically bind to the colorectal tumor rather than other normal organs, such as esophagus, stomach, cecum and normal colon tissue (Figure 5). These results proved that ^111^In-cetuximab possesses high specificity to EGF receptor over-expressed tumor cells, which was also observed in the former repor. [19, 20] Therefore, it was suggested that ^111^In-cetuximab could be applied to detect human metastatic colorectal cancer.

As shown in the nano-SPECT/CT and autoradiography images of ^111^In-cetuximab in the HCT-116/Luc metastatic colorectal tumors, compared with ^111^In-DTPA, there was higher uptake of ^111^In-cetuximab at 48 and 72 hours. In addition, the biodistribution of ^111^In-cetuximab and ^111^In-DTPA in HCT-116/Luc metastatic colorectal tumor showed that ^111^In-cetuximab could remain in the tumor and increase with time, but relatively low in the other organs.

In our studies, the spleen uptake of ^111^In-cetuximab was significantly higher at 72 hours than at 24 hours (Figure 6). That's because cetuximab is a monoclonal antibody with molecular weight about 150 kDa, which resulting that this macromolecule could been filtered by reticuloendothelial system (like spleen and liver). [19, 20] Hence, the spleen uptake of ^111^In-cetuximab was increased until 72 hours. Therefore, the ^111^In-cetuximab would be an effective diagnostic agent and clearly detect EGF receptor-highly expressed metastatic colon tumors even tumors were in the abdominal cavity.

Figure 7 showed that kidney had higher signal at 24 hours after injection of ^111^In-DTPA comparing with 72 hours (*P* < 0.05). The results proved that the ^111^In-DTPA metabolism through the urinary system. The ^111^In-DTPA were accumulated to tumors at 24 hours after injection, but reduced significantly at 72 hours. In addition, comparing with ^111^In-DTPA, the ^111^In-cetuximab can be continuously accumulated in the tumor from 24 to 72 hours after injection as shown in Figure 7.

In conclusion, we suggested that ^111^In-cetuximab had a high specificity to target EGF receptor overexpression cells in the human metastatic tumor-bearing and *in situ* colorectal tumor-bearing mice models. This research proved cetuximab could effectively target EGF receptor. In clinical, the ^111^In-cetuximab will be a potential and easier tool for detecting EGF receptor expression in human metastatic colorectal carcinoma.

## MATERIALS AND METHODS

### Cell lines

HCT-116, HCT-116/Luc, HT-29, SW-620 and CT-26 the five cell lines were used for *in vitro* and/or *in vivo* studies. They were obtained from BCRC (Bioresorce Collection and Research Center) of FIRDI (Food Industry Research and Development Institute). Cells were grown in Mccoy's 5A modified medium (HCT-116 and HCT-116/Luc), RPMI-1640 medium (HT-29) and high glucose Dulbecco's modified Eagle's medium (SW-620, CT-26) supplemented with 10% fetal bovine serum (FBS) and 1% P/S (Penicillin/Streptomycin solution) at 37°C in incubator containing 5% CO_2_.

### Cellular uptake of cetuximab

The monolayer of HCT-116, HT-29 and SW-620 cells was washed with PBS and incubated with trypsin-EDTA at 37°C for 5 min. The cells were collected and re-suspended in medium. Survival of all cells was counted using a trypan-blue dye exclusion assay. The cell number in the suspension was adjusted, and 1 × 10^6^ cells in 100 μl of medium were used for the cellular uptake test. After incubating the samples with PE-conjugated mouse anti-human EGF receptor antibody (BD Pharmingen) for 20 min at 37°C in a humidified incubator (5% CO_2_), the cells were washed twice with 3 mL of PBS to remove unbound antibody by centrifugation for 5 min. The cell suspensions were analyzed immediately by the flow cytometry.

HCT-116, HT-29 and SW-620 cells were lysed in RIPA buffer. The cell lysates were centrifuged at 12, 000 rpm for 15 minutes to pellet the cell debris. The cleared lysates (containing 30 μg of protein) were separated on 8% SDS polyacrylamide gels and transferred onto polyvinylidene difluoride membranes (Millipore). After blocking, the membranes were incubated with polyclonal antibody against human EGF receptor (Santa Cruz Biotechnology) at 4°C overnight, followed by a second antibody labeled with horseradish peroxidase (HRP), and developed with enhanced chemiluminescence (ECL) reagents (Santa Cruz Biotechnology). The signals on the blots were scanned and analyzed using the UVP imaging system (BioSpectrum® Imaging System). Each value is shown as an arbitrary unit (AU), and is representative of at least two independent experiments.

### ^111^In-cetuximab synthesis

Cetuximab was conjugated with DTPA to produce DTPA-cetuximab. ^111^InCl_3_ (370 MBq) was diluted in 100 μL of 0.2 M sodium acetate buffer (pH 5.5) and add to 100 μg of DTPA-cetuximab. The reaction mixture was incubated for 1 hour at 37°C with constant shaking. ^111^In-cetuximab was generated at an average yield of ~ 80%, and the radiochemical purity was > 85% in this study.

### Animal models

We used six to eight weeks old nude mice that were xenografted subcutaneously in the right and left hind leg with the human HCT-116 and SW-620 cell lines (3 × 10^6^ cells), respectively. *In situ* colorectal tumor of CT-26 cell (3 × 10^5^ cells) was xenografted into rectum of mice by small animal endoscopy. In addition, metastatic colorectal tumors was xenografted HCT-116/Luc cells (3 × 10^6^ cells) into abdominal of nude mice. Tumors with a mean volume of 100–300 mm^3^ were used in the experiments. Animals were housed in filter-topped cages in a specific-pathogen-free unit in accordance with INER institutional guidelines.

The metastatic colorectal tumor-bearing nude mice were were injected Luciferin with an IVIS imaging system (Xenogen, Alameda, CA, USA) for detection the tumor.

### Nano-SPECT/CT scintigraphy

Nano single-photon emission computed tomography (SPECT)/computed tomography (CT) was also performed to evaluate the distribution of the ^111^In-cetuximab and/or ^111^In-DTPA in the mice bearing subcutaneously, orthotropic and metastatic tumors by nano-SPECT/CT. SPECT images and X-ray CT images were acquired using a nano-SPECT/CT scanner system (Bioscan Inc., USA.).

HCT-116 and SW-620 cells bearing subcutaneous colon tumors was used to detect ^111^In-cetuximab by nano-SPECT/CT. Aim of this study was for select which EGF receptor expression cell bearing colon tumor could had more binding to ^111^In-cetuximab. Subcutaneous tumor mice were received an intravenous injection of ^111^In-cetuximab (equivalent to 22.2 MBq), when tumor volume up to 300 mm^3^. The nano-SPECT/CT images were collected at post injection 1, 4, 24 and 48 hours.

*In vivo* CT-26 cell bearing orthotropic tumor image and *ex vivo* nano-SPECT/CT image of ^111^In-cetuximab were obtained at 72 h intravenous on days 9 *in situ* colorectal tumor.

HCT-116/Luc bearing metastatic colorectal tumors mice received an intravenous injection of ^111^In-cetuximab and ^111^In-DTPA, equivalent to 22.2 MBq of ^111^In, when tumors reached a volume of 5 to 60 mm^3^. The distribution of ^111^In-cetuximab in the mice bearing HCT-116/Luc adenocarcinoma tumors was evaluated by nano-SPECT/CT images at 1, 4, 24, 48 and 72 hours after the ^111^In-cetuximab and ^111^In-DTPA were intravenously injected.

### Autoradiography

After the nano-SPECT/CT studies, HCT-116 bearing subcutaneous tumor nude that had been injected ^111^In-cetuximab after 48 hours. Whole body of nude mice were collected, frozen, and embedded in OCT compound (Tissue Tek, Sakura, Torrance, CA). Frozen sections were placed in contact with a BASMS 2040 imaging plate (Fujifilm, Japan) for thirty days. After complete exposure, the imaging plate was analyzed with an FLA-5100 reader (Fujifilm, Japan) and Multi Gauge V3.0 software (Fujifilm, Japan).

HCT-116/Luc bearing abdominal nude mice whole body of ^111^In-cetuximab and ^111^In-DTPA after injected 72 hours were collected, frozen, and embedded in OCT compound after the nano-SPECT/CT studies. The follow steps described as above paragraph.

### Biodistribution

The biodistribution of ^111^In-cetuximab and ^111^In-DTPA was studied by injecting intravenously through a tail vein of HCT-116/Luc bearing metastatic colon tumor mice. The tumor-bearing mice were sacrificed at 24, and 72 hours after the ^111^In-cetuximab and ^111^In-DTPA were injected, and then the blood, smooth, lung, heart, liver, spleen, kidney, muscle, small intestine, urinary bladder, bone, brain and tumors were collected, and the uptake of radioactivity was measured by a gamma counter. Tissue distribution data were expressed as the percentage of injected dose per gram tumor tissue (ID%/g).

### Immunohitochemical staining

After ^111^In-cetuximab autoradiography of metastasis abdominal colon tumor, the tumors were taken out from mice, then sections. In staining procedure, the slides were fixed in acetone at 4°C for 10 minutes. Rabbit anti EGF receptor primary antibody (Anti-EGFR affinity isolated antibody, SIGMA, China) was used to detect EGF receptor expression incubation at 4°C overnight, and then washed and used broad secondary antibody for 20 minutes. Next washed and used HRP for 10 minutes. Finally, washed and DAB was used for 3 minutes for visualization.

### Statistics

All data are expressed as mean ± standard deviation. The significance of difference in this study between ^111^In-Cetuximab and ^111^In-DTPA two groups was analyzed by the *t*-test. Result value of *P* < 0.05 was considered as statistically significant between experiment group and control group.
